# Improving yeast strains using recyclable integration cassettes, for the production of plant terpenoids

**DOI:** 10.1186/1475-2859-10-4

**Published:** 2011-01-28

**Authors:** Codruta Ignea, Ivana Cvetkovic, Sofia Loupassaki, Panagiotis Kefalas, Christopher B Johnson, Sotirios C Kampranis, Antonios M Makris

**Affiliations:** 1 Department of Natural Products and Biotechnology, Centre International de Hautes Etudes Agronomiques Méditerranéennes. Mediterranean Agronomic Institute of Chania, P.O. Box 85, Chania 73100, Greece; 2 Department of Food Quality and Chemistry of Natural Products, Centre International de Hautes Etudes Agronomiques Méditerranéennes. Mediterranean Agronomic Institute of Chania, P.O. Box 85, Chania 73100, Greece; 3 Department of Biology, University of Crete, Vasilika Vouton, 71409, Heraklio, Crete, Greece; 4 Institute of Agrobiotechnology/CERTH, P.O.Box 60361, Thermi 57001, Thessaloniki, Greece

## Abstract

**Background:**

Terpenoids constitute a large family of natural products, attracting commercial interest for a variety of uses as flavours, fragrances, drugs and alternative fuels. *Saccharomyces cerevisiae *offers a versatile cell factory, as the precursors of terpenoid biosynthesis are naturally synthesized by the sterol biosynthetic pathway.

**Results:**

*S. cerevisiae *wild type yeast cells, selected for their capacity to produce high sterol levels were targeted for improvement aiming to increase production. Recyclable integration cassettes were developed which enable the unlimited sequential integration of desirable genetic elements (promoters, genes, termination sequence) at any desired locus in the yeast genome. The approach was applied on the yeast sterol biosynthetic pathway genes *HMG2*, *ERG20 *and *IDI1 *resulting in several-fold increase in plant monoterpene and sesquiterpene production. The improved strains were robust and could sustain high terpenoid production levels for an extended period. Simultaneous plasmid-driven co-expression of *IDI1 *and the *HMG2 *(K6R) variant, in the improved strain background, maximized monoterpene production levels. Expression of two terpene synthase enzymes from the sage species *Salvia fruticosa *and *S. pomifera *(SfCinS1, SpP330) in the modified yeast cells identified a range of terpenoids which are also present in the plant essential oils. Co-expression of the putative interacting protein HSP90 with cineole synthase 1 (SfCinS1) also improved production levels, pointing to an additional means to improve production.

**Conclusions:**

Using the developed molecular tools, new yeast strains were generated with increased capacity to produce plant terpenoids. The approach taken and the durability of the strains allow successive rounds of improvement to maximize yields.

## Background

**Figure 1:**
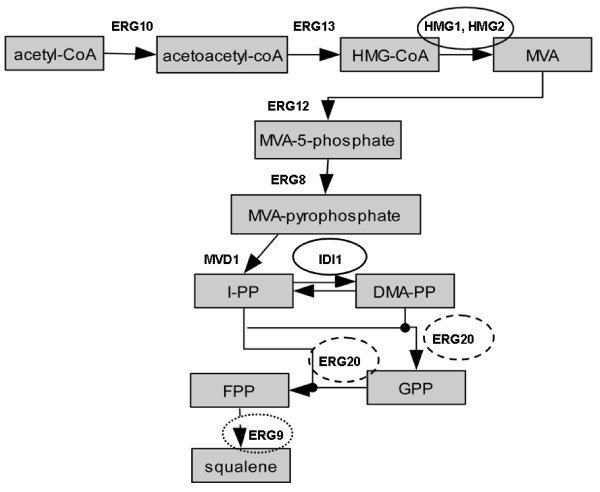
**The Mevalonate pathway of GPP and FPP biosynthesis in yeast**. The non-boxed rectangles show the points of integration of the introduce plant enzymes producing monoterpenes and sesquiterpenes. The encircled enzymatic steps represent targets of modification. Solid lines represent introduction of extra gene copies (*HMG2*, *IDI1*), broken lines show the introduction of a strong inducible promoter in one of two alleles, and in dotted line is the deletion of one allele to generate a haploinsufficient strain.

An important class of secondary metabolites, terpenoids and isoprenoids contribute more than 50,000 compounds to the rich chemical diversity of natural product structures [1]. Many of them have attracted commercial interest for their medicinal properties or as flavour and fragrance additives in the food and cosmetic industry. Among them taxol, a diterpene from yew, has successfully been established in the clinic as a major antineoplastic agent, artemisinin is an effective antimalarial agent and sclareol is industrially important diterpene used by the fragrance industry [2-4]. Recently, attention has also focused on microbial produced terpenes as biodiesel [5,6] All terpenoids are biosynthesized from two C_5 _precursors, isopentenyl diphosphate (IPP) and dimethylallyl diphosphate (DMAPP) [7]. Two distinct and independent biosynthetic routes to IPP formation exist. In yeast and mammals IPP originates from acetyl-CoA through the intermediate mevalonic acid (MVA) (Figure 1). In eubacteria and plastid associated isoprenoids in algae and plants, IPP is derived by the condensation of pyruvate and glyceraldehyde-3-phosphate, via the 1-deoxyxylulose-5-phosphate (DXP) pathway. IPP then gives rise through the action of prenyltransferase enzymes to the higher order building blocks, geranyl pyrophosphate (GPP; C_10_), farnesyl pyrophosphate (FPP; C_15_) and geranylgeranyl pyrophosphate (GGPP; C_20_) [7-9]. Monoterpenes (C_10_) are known to play important chemoecological roles in the interactions between plants and their environments, often playing a protective role against herbivores and pathogens [10]. Many plant species, including *Salvia *sp. (sage), produce and store large amounts of terpenoid-rich resins and essential oils in differentiated tissues such as glandular trichomes found on the surface of plant aerial parts [11,12]. Biosynthesis of monoterpenes and sesquiterpenes has previously been examined in the secretory cells of peppermint (*Mentha × piperita*), an essential oil-producing plant that belongs to the large *Lamiaceae *family (which also includes sage and basil). Monoterpene biosynthesis and accumulation was indeed shown to localize in the glandular trichomes [12,13].

*Saccharomyces cerevisiae *is a unicellular eukaryotic organism traditionally regarded as a model system for investigating cellular physiology and widely used as a cell factory for biotechnological applications. Strain improvement has relied in the past on random mutagenesis and classical genetic approaches. In recent years, developments in molecular genetics have made it possible to introduce specific genetic perturbations by modifying the promoter strength of a given gene, to perform gene deletions, or to introduce whole new genes or biosynthetic pathways. Metabolic engineering of an amenable organism such as *S. cerevisiae *aiming to direct cellular resources and the biosynthetic machinery towards the production of highly desirable natural products, can be an environmentally friendly and economically viable approach to the production of high-added value compounds [14]. *S. cerevisiae *offers a unique advantage for the production of terpenoids, as the precursors of terpene biosynthesis (GPP, FPP and GGPP) are naturally synthesized by the sterol biosynthetic pathway. Recent efforts in yeast metabolic engineering for the production of terpenes have pointed to a significant potential for improvement in the yields of compounds produced [15-19].

In the current work we aimed to improve yeast strains in a targeted way while avoiding to introduce bacterial plasmid sequences into the yeast genome. The approach was based on recyclable cassette vectors that enable unlimited modifications of the genome. Through the use of the cre-lox recombination system the selection markers can be excised and reused. We employed this approach to introduce strong inducible promoter upstream of endogenous yeast genes and to incorporate at any desired genetic locus extra copies of genes controlled by strong promoters. When applied to the terpenoid biosynthetic pathway, this approach afforded yeast stains which exhibit several fold increase in the yield of monoterpene and sesquiterpene production.

## Results

### Selection of an optimal yeast strain for terpenoid production

**Figure 2:**
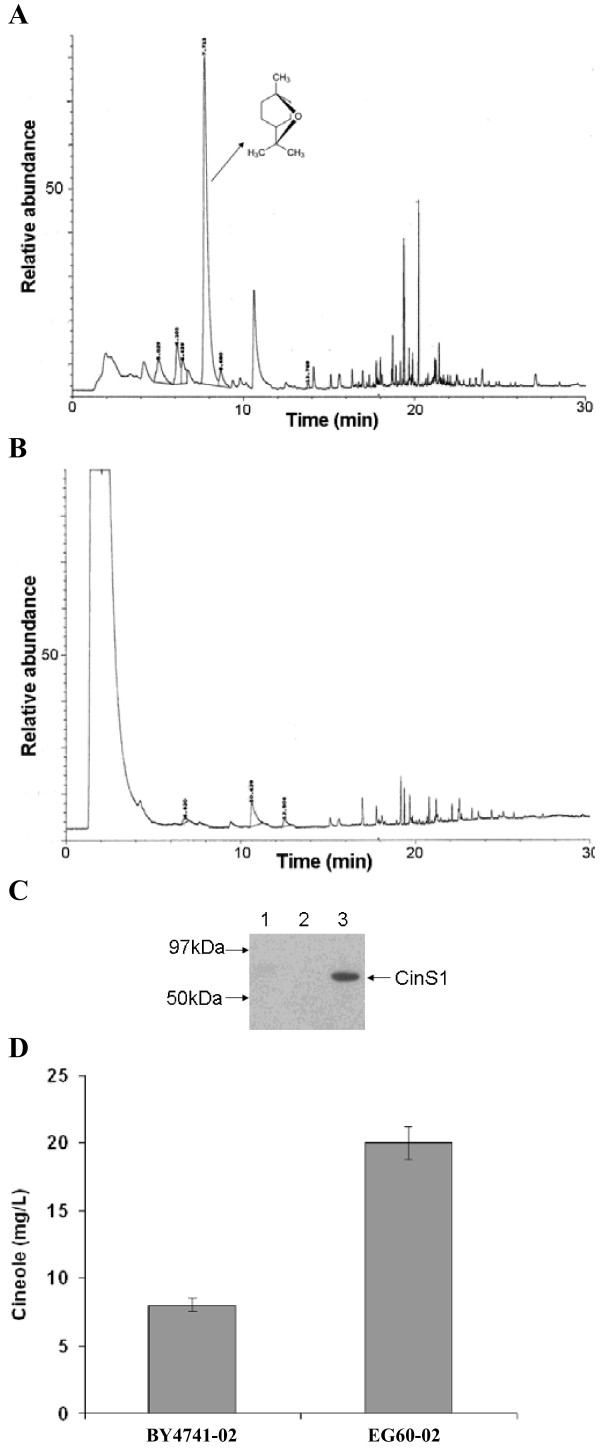
**Production of cineole in yeast**. (A) EG60-01 yeast cells expressing SfCinS1, under the control of the galactose inducible promoter, were sampled by SPME and analyzed by gas chromatography. (B) EG60-06 cells harboring empty vector do not produce cineole. (C) Western blot showing expression of SfCinS1, lane 1 protein extracts from EG60 cells, lane 2 protein extracts from EG60-01 cells harboring the SfCinS1 grown in glucose, and in lane 3 grown in galactose, which induces protein expression. (D) Quantification of cineole production, in BY4741-02 cells (reference strain) versus EG60-02 cells.

Yeast laboratory and industrial strains are known to possess a high degree of phenotypic variability towards adverse growth conditions such as thermal stress, oxidative stress and nutrient limitation. To initiate our yeast improvement effort for the production of terpenoids, we selected to test a strain (EG60, Table 1) for which there was evidence of increased sterol production (F. Mantzouridou, unpublished data). The plasmid pJG4-6/SfCinS1, which expresses inducibly the *S. fruticosa *(Greek sage) cineole synthase 1 fused to a hemaglutinin tag (HA), was introduced into EG60 cells and the standard reference laboratory strain BY4741 (Table 1). Expression of the SfCinS1 was achieved by switching cells in galactose-based media, and the presence of the protein was confirmed in western blots using antibodies against HA (Figure 2C). For the subsequent experimental work, a non-tagged version of SfCinS1 was used to avoid any interference of the tag to enzyme activity, as monoterpene synthases are sensitive to alterations in the N-terminus (data not shown). Equal amounts of yeast cells were induced in galactose, and were subsequently transferred in buffer solution. Sampling of the cells in buffer tended to yield lower background volatiles. At 24 hours after enzyme induction the ambient atmosphere of the culture flasks was sampled by inserting a Solid Phase Microextraction (SPME) fiber for 30 min. and the adsorbed volatiles were analyzed by Gas Chromatography-Mass Spectrometry (GC-MS). As anticipated, cells harboring the SfSinS1 construct produced cineole (Figure 2A), whereas cells with empty vector yielded no significant terpenoids (Figure 2B). EG60-01 cells expressing SfCinS1 produced 2.5-fold higher levels of cineole compared to BY4741-01 cells (Table 1), confirming the initial hypothesis that strains with increased sterol levels would produce higher terpene yields (Figure 2D).

**Table 1:** Yeast strains used in this study

Strain	Genotype	Plasmid description	Source
BY4741-01	Mat a, *his3*Δ*1, leu2*Δ*0, met15*Δ*0, ura3*Δ*0.*	pJG4-6/SfCinS1 2μ *TRP1 *P_Gal1_-HA-SfCinS1	This study
BY4741-02	Mat a, *his3*Δ*1, leu2*Δ*0, met15*Δ*0, ura3*Δ*0.*	pJG4-4/SfCinS1 2μ *TRP1 *P_Gal1_-SfCinS1	This study
BY4741-04	Mat a, *his3*Δ*1, leu2*Δ*0, met15*Δ*0, ura3*Δ*0.*	pYESmyc/P330 2μ *URA3 *P_Gal1_-myc-P330	This study
EGY48	Mat α, *ura3, trp1, his3*, 6xLexA operators::*LEU2*. Derivative of the U457.		Erica Golemis
EGY48-01	Mat α, *ura3, trp1, his3*, 6xLexA operators::*LEU2*. Derivative of the U457.	pYES/SfCinS1-LexA 2μ *URA3 *P_Gal1_-SfCinS1-LexA	This study
EGY48-02	Mat α, *ura3, trp1, his3*, 6xLexA operators::*LEU2*. Derivative of the U457.	pYES-LexA 2μ *URA3 *P_Gal1_-LexA	This study
EG60	Mat α, *ura3, trp1, his3, leu2.*		Erica Golemis
EG60-01	Mat α, *ura3, trp1, his3, leu2*	pJG4-6/SfCinS1 2μ *TRP1 *P_Gal1_-HA-SfCinS1	This study
EG60-02	Mat α, *ura3, trp1, his3, leu2.*	pJG4-4/SfCinS1 2μ *TRP1 *P_Gal1_-SfCinS1	This study
EG60-03	Mat α, *ura3, trp1, his3, leu2*	pYESmyc/P330 2μ *URA3 *P_Gal1_-myc-P330	This study
EG60-04	Mat α, *ura3, trp1, his3, leu2*	pJG4-4/SfCinS1 2μ *TRP1 *P_Gal1_-SfCinS1, pYESmyc/IDI1 2μ *URA3 *P_Gal1_-myc-IDI1	This study
EG60-05	Mat α, *ura3, trp1, his3 leu2*	pJG4-4/SfCinS1 2μ *TRP1 *P_Gal1_-SfCinS1, pYES 2μ *URA3 *P_Gal1_	This study
EG60-06	Mat α, *ura3, trp1, his3 leu2*	pJG4-4/SfCinS1 2μ *TRP1 *P_Gal1_-SfCinS1, pYES/HSP90 2μ *URA3 *P_Gal1_-HSP90	This study
EG60-07	Mat α, *ura3, trp1, his3 leu2*	pJG4-4 2μ *TRP1 *P_Gal1 _- empty vector	
EG61	Mat a, *ura3, trp1, his3 leu2*		This study
KSY10	Mat a, P_Gal1_-*HMG2 *(K6R)::*URA3, trp1, his3, leu2*		This study
AM62	Mat α, Δ*erg6*:: HIS5, *ura3, trp1, his3, leu2 *Derivative of EG60.		This study
AM63	Mat α, P_Gal1_-*HMG2 *(K6R)::*URA3, trp1, his3, leu2*.		This study
AM63-01	Mat α, P_Gal1_-*HMG2 *(K6R)::*URA3, trp1, his3, leu2*.	pJG4-4/SfCinS1 2μ *TRP1 *P_Gal1_-SfCinS1	This study
AM63-02	Mat α, P_Gal1_-*HMG2 *(K6R)::*URA3, trp1, his3, leu2*.	pJG4-4/SfCinS1 2μ *TRP1 *P_Gal1_-SfCinS1, pYX143/GDS 2μ *LEU2 *P_TPI1_-GDS	This study
AM64	Mat α, *URA3*-*hisG*-P_Gal1_-*HMG2 *(K6R)::HO, *trp1, his3*, *leu2*, Δ*erg6*::HIS5. Derivative of AM63.		This study
AM65	Mat α, P_Gal1_-*HMG2 *(K6R), *ura3, trp1, his3, leu2*. Derivative of AM64.		This study
AM65-01	Mat α, P_Gal1_-*HMG2 *(K6R), *ura3, trp1, his3, leu2.*	pJG4-4/SfCinS1 2μ *TRP1 *P_Gal1_-SfCinS1,	This study
AM65-02	Mat α, P_Gal1_-*HMG2 *(K6R), *ura3, trp1, his3,leu2.*	pJG4-4/SfCinS1 2μ *TRP1 *P_Gal1_-SfCinS1, pYESmyc/IDI1 2μ *URA3 *P_Gal1_-myc-*IDI1*	
AM66	Mat α/a, P_Gal1_-*HMG2 *(K6R) X2::HO, *ura3, trp1, his3, leu2*. Derivative of AM63 and KSY10 back-crossed 4 times.		This study
AM66-01	Mat α/a, P_Gal1_-*HMG2 *(K6R) x2::HO, *ura3, trp1, his3, leu2*.	pJG4-4/SfCinS1 2μ *TRP1 *P_Gal1_-SfCinS1	This study
AM66-02	Mat α/a, P_Gal1_-*HMG2 *(K6R) x2::HO, *ura3, trp1, his3*, *leu2*	pYESmyc/P330 2μ *URA3 *P_Gal1_-myc-P330	This study
AM66-03	Mat α/a, P_Gal1_-*HMG2 *-(K6R) x2::HO, *ura3, trp1, his3*, *leu2*.	pJG4-4/SfCinS1 2μ *TRP1 *P_Gal1_-SfCinS1, pYESmyc/IDI1 2μ *URA3 *P_Gal1_-myc-*IDI1*	This study
AM67	Mat α/a, P_Gal1_-*HMG2 *(K6R) x2, P_Gal1_-HA-*ERG20*-*URA3*, *trp1, his3, leu2*. Derivative of AM66.		This study
AM67-01	Mat α/a, P_Gal1_-*HMG2 *(K6R)x2, P_Gal1_-HA-*g*-URA3, *trp1, his3, leu2.*.	pB227/GAL-cre, CEN *LEU2*	This study
AM67-02	Mat α/a, P_Gal1 _-*HMG2 *(K6R) x2, P_Gal1_-HA-*ERG20-URA3*, *trp1, his3*, *leu2*	pJG4-4/SfCinS1 2μ *TRP1 *P_Gal1_-SfCinS1	This study
AM68	Mat α/a, P_Gal1_-*HMG2 *-(K6R) x2, P_Gal1_-HA-*ERG20*, *ura3, trp1, his3*. Derivative of AM67.		This study
AM68-01	Mat α/a, P_Gal1_-*HMG2 *(K6R) x2, P_Gal1_-HA-*ERG20*, *ura3, trp1, his3*, *leu2*	pJG4-4/SfCinS1 2μ *TRP1 *P_Gal1_-SfCinS1	This study
AM68-02	Mat α/a, P_Gal1_-*HMG2 *-(K6R) x2, P_Gal1_-HA-*ERG20*, *ura3, trp1, his3*, *leu2*	pYESmyc/P330 2μ *URA3 *P_Gal1_-myc-P330	This study
AM68-03	Mat α/a, P_Gal1_-*HMG2 *(K6R) x2, P_Gal1_-HA-*ERG20*, *ura3, trp1, his3*, *leu2*	pJG4-4/SfCinS1 2μ *TRP1 *P_Gal1_-SfCinS1, pYESmyc/IDI1 2μ URA3 P_Gal1_-myc-*IDI1*	This study
AM68-04	Mat α/a, P_Gal1-_*HMG2 *(K6R) x2, P_Gal1_-HA-*ERG20*, *ura3, trp1, his3*, *leu2*	pYESmyc/P330 2μ *URA3 *P_Gal1_-myc-P330, pYIC1/IDI1 2μ LEU2 P_Gal1_-*IDI1*	This study
AM69	Mat α/a, P_Gal1_-*HMG2 *-(K6R)x2, P_Gal1_-*ERG20*-*URA3*, *trp1, his3*, *leu2*, Δ*erg9*:: HIS5. Derivative of AM67.		This study
AM70	Mat α/a, P_Gal1_-HMG2 -(K6R) x2, P_Gal1_-HA-*ERG20*, *ura3, trp1, his3*, *leu2*, Δ*erg9*:: HIS5. Derivative of AM68.		This study
AM70-01	Mat α/a, P_Gal1_-*HMG2 *(K6R) x2, P_Gal1_-HA-*ERG20*, *ura3, trp1, his3*, *leu2*, Δ*erg9*:: HIS5.	pJG4-4/SfCinS1 2μ *TRP1 *P_Gal1_-SfCinS1	This study
AM70-02	Mat α/a, P_Gal1_-HMG2 -(K6R) x2, P_Gal1_-HA-*ERG20*, *ura3, trp1, his3*, *leu2*, Δ*erg9*:: HIS5.	pYESmyc/P330 2μ *URA3 *P_GAL1_-myc-P330	This study
AM70-03	Mat α/a, P_Gal1_-*HMG2 *(K6R) x2,P_Gal1_-HA-*ERG20*, *ura3, trp1, his3, leu2*, Δ*erg9*:: HIS5.	pJG4-4/SfCinS1 2μ *TRP1 *P_Gal1_-SfCinS1, pYESmyc/IDI1 2μ *URA3 *P_Gal1_-myc-IDI1	This study
AM70-04	Mat α/a, P_Gal1_-*HMG2 *(K6R) x2,P_Gal1_-HA-*ERG20*, *ura3, trp1, his3, leu2*, Δ*erg9*:: HIS5.	pYESmyc/P330 2μ *URA3 *P_GAL1_-myc-P330, pYIC1/IDI1 2μ *LEU2 *P_Gal1_-*IDI1*	This study
AM74	Mat α/a, P_Gal1_-*HMG2 *(K6R)x2::HO, P_Gal1_-HA-*ERG20*, P_Adh1_-myc:*:tHMG1*, *URA3, trp1, his3, leu2*. Derivative of AM68.		This study
AM75	Mat α/a, P_Gal1_-*HMG2 *(K6R) x2::HO, P_Gal1_-HA-*ERG20*, P_Adh1_-myc::*tHMG1*, URA3, *trp1, his3*, *leu2*, Δ*erg9*::HIS5. Derivative of AM70.		This study
AM76	Mat α/a, P_Gal1_-*HMG2 *(K6R) x2::HO, *ura3, trp1, his3, leu2*, Δ*erg9*:: HIS5. Derivative of AM66.		This study
AM76-01	Mat α/a, P_Gal1_-*HMG2 *(K6R) x2::HO, *ura3, trp1, his3, leu2*, Δ*erg9*:: HIS5.	pJG4-4/SfCinS1 2μ *TRP1 *P_Gal1_-SfCinS1, pYESmyc/IDI1 2μ URA3 P_Gal1_-myc-*IDI1*	This study
AM77	Mat α, P_Gal1_-*HMG2 *(K6R), *ura3, trp1, his3, leu2*:: P_Gal1_-*IDI1*-HIS5. Derivative of AM65.		This study
AM77-01	Mat α, P_Gal1_-*HMG2 *(K6R), *ura3, trp1, his3, leu2*:: P_Gal1_-*IDI1*-HIS5.	pB227/GAL-cre, CEN LEU2	This study
AM77-02	Mat α, P_Gal1_-*HMG2 *(K6R), *ura3, trp1, his3, leu2*:: P_GAL1_-*IDI1*-HIS5.	pJG4-4/SfCinS1 2μ *TRP1 *P_Gal1_-SfCinS1	This study
AM78	Mat α, P_Gal1_-*HMG2 *(K6R), *ura3, trp1, his3, leu2*:: P_Gal1_-*IDI1*. Derivative of AM77.		This study
AM78-01	Mat α, P_Gal1_-*HMG2 *(K6R), *ura3, trp1, his3, leu2*:: P_Gal1_-*IDI1*.	pJG4-4/SfCinS1 2μ *TRP1 *P_Gal1_-SfCinS1	This study
AM78-02	Mat α, P_Gal1_-*HMG2 *(K6R), *ura3, trp1, his3, leu2*:: P_Gal1_-*IDI1*.	pJG4-4/SfCinS1 2μ *TRP*1 P_Gal1_-SfCinS1, pYESmyc/IDI1 2μ *URA3 *P_Gal1_-myc-*IDI1*	This study
AM78-03	Mat α, P_Gal1_-*HMG2 *(K6R), *ura3, trp1, his3, leu2*:: P_Gal1_-*IDI1*.	pJG4-4/SfCinS1 2μ *TRP1 *P_Gal1_-SfCinS1, pYESmyc/IDI1 2μ *URA3 *P_Gal1_-myc-*IDI1*, pYIC1 2μ *LEU2 *P_Gal1_	This study
AM78-03	Mat α, P_Gal1_-*HMG2 *(K6R), *ura3, trp1, his3, leu2*:: P_Gal1_-*IDI1*.	pJG4-4/SfCinS1 2μ *TRP1 *P_Gal1_-SfCinS1, pYESmyc 2μ *URA3 *P_Gal1_-myc, pYIC1/IDI1 2μ *LEU2 *P_Gal1_-HA-*IDI1*	This study
AM78-04	Mat α, P_Gal1_-*HMG2 *(K6R), *ura3, trp1, his3, leu2*:: P_Gal1_-*IDI1*.	pJG4-4/SfCinS1 2μ *TRP1 *P_Gal1_-SfCinS1, pYIC1/IDI1 2μ *LEU2 *P_Gal1_-HA-*IDI1*	This study
AM78-05	Mat α, P_Gal1_-*HMG2 *(K6R), *ura3, trp1, his3, leu2*:: P_Gal1_-*IDI1*.	pJG4-4/SfCinS1 2μ *TRP1 *P_Gal1_-SfCinS1, pYESmyc/IDI1 2μ URA3 P_Gal1_-myc-*IDI1*, pYIC1/IDI1 2μ *LEU2 *P_Gal1_-HA-*IDI1*	This study
AM78-06	Mat α, P_Gal1_-*HMG2 *(K6R), *ura3, trp1, his3, leu2*:: P_Gal1_-*IDI1*.	pJG4-4/SfCinS1 2μ *TRP1 *P_Gal1_-SfCinS1, pYESmyc 2μ *URA3 *P_Gal1_-myc-, pYIC1/HMG2(K6R) 2μ *LEU*2 P_Gal1_-HA-*HMG2 *(K6R)	This study
AM78-07	Mat α, P_Gal1_-*HMG2 *(K6R), *ura3, trp1, his3, leu2*:: P_Gal1_-*IDI1*.	pJG4-4/SfCinS1 2μ *TRP1 *P_Gal1_-SfCinS1, pYESmyc/IDI1 2μ *URA3 *P_Gal1_-myc-*IDI1*, pYIC1/HMG2(K6R) 2μ *LEU*2 P_Gal1_-HA-*HMG2 *(K6R)	This study
AM78-08	Mat α, P_Gal1_-*HMG2 *(K6R), *ura3, trp1, his3, leu2*:: P_Gal1_-*IDI1*.	pJG4-4/SfCinS1 2μ TRP1 P_Gal1_-SfCinS1, pYES 2μ *URA3 *P_Gal1_	This study
AM78-09	Mat α, P_Gal1_-*HMG2 *(K6R), *ura3, trp1, his3, leu2*:: P_Gal1_-*IDI1*.	pJG4-4/SfCinS1 2μ TRP1 P_Gal1_-SfCinS1, pYES/HSP90 2μ *URA3 *P_Gal1_-HSP90	This study
AM78-10	Mat α, P_Gal1_-*HMG2 *(K6R), *ura3, trp1, his3, leu2*:: P_Gal1_-*IDI1*.	pJG4-4/SfCinS1 2μ *TRP1 *P_Gal1_-SfCinS1, pYIC1/HMG2(K6R) 2μ *LEU2 *P_Gal1_-HA-*HMG2 *(K6R)	This study

### Integration of a K6R variant of HMG2 into the genome

**Figure 3:**
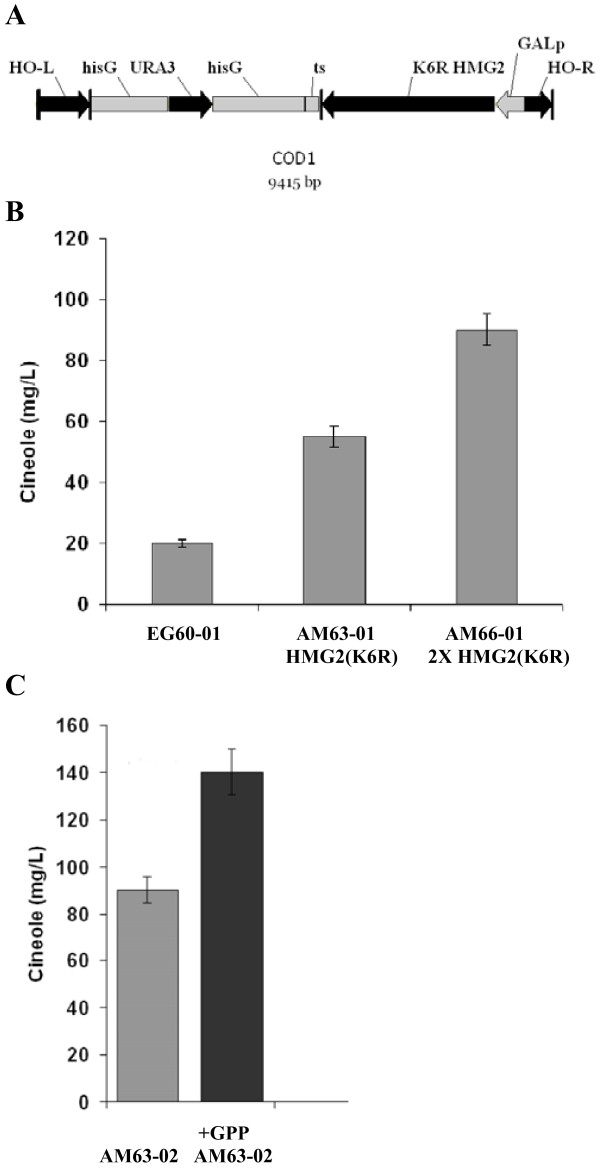
**Integration of HMG2(K6R) variant leads to cineole production increase**. (A) Map of the homologous recombination cassette designed to integrate a stabilized copy of HMG2(K6R) into the HO locus under the control of the galactose promoter. The hisG-URA3-hisG part of the cassette is subsequently excised from the genome by plating in FOA plates; (B) Cineole production in AM63 and AM65 cells; (C) Cineole yields in AM63 cells co-expressing the *P. abies *geranyl pyrophosphate synthase (GDS). Exogenous GPP was also added to assess the level of saturation of the system.

To improve terpene production by the EG60 strain, we sought to incorporate alterations in the sterol biosynthetic pathway stably into the chromosomal genome avoiding the integration of exogenous bacterial plasmid sequences. As the first step in this approach, the previously developed vector (M4366), containing the *HO*-*hisG*-*URA3*-*hisG*-poly-*HO *cassette, was employed [20]. The vector is designed to enable integration by homologous recombination at the HO locus, which in the vast majority of laboratory yeast strains is already mutated [20,21]. A cassette composed of the galactose promoter, a multicloning site and the terminator sequence was inserted into the vector's multicloning site. The first gene chosen in the pathway was *HMG2*, which encodes an enzyme responsible for converting 3-hydroxy-3-methylglutaryl (HMG)-CoA into mevalonic acid (Figure 1) [22]. HMGR is a key enzyme in sterol biosynthesis and is subject to feedback regulation in yeasts, plants and animals [23]. Yeast has 2 isozymes of HMGR termed hmg1p and hmg2p and encoded by *HMG1 *and *HMG2 *genes respectively. Mutation of lysine 6 into arginine in *HMG2 *is known to stabilize the protein from protein degradation [24]. The variant K6R *HMG2 *was inserted at the multicloning site of the M4366-P_Gal1 _resulting in the HOR-P_Gal1_-*HMG2 *(K6R)-ts-hisG-*URA3*-hisG-HOL DNA fragment (COD1; Figure 3A). COD1 was then used to transform EG60 cells, creating strain AM63. Colonies which were capable of growing in media lacking uracil were selected and tested by PCR for proper integration. The hisG-*URA3*-hisG cassette was excised by inducing recombination in the AM63 strain between the hisG repeats flanking the URA3 gene, leaving behind the P_Gal1_-*HMG2 *(K6R)-ts, thus generating strain AM65 (Table 1). The pJG4-4/SfCinS1 plasmid was introduced into the AM63 strain and cells were tested for cineole production (AM63-01; Table1). A reproducible 2.8-fold increase in cineole production compared to the parental strain EG60-02 (Table 1) was observed (Figure 3B). Conversion of the strain into a diploid homozygous strain for the P_Gal1_-*HMG2 *(K6R) AM66 (Table 1) led to an additional production boost (Figure 3B). To assess whether additional improvements in cineole production could be attained, the *Picea abies *geranyl diphosphate synthase (GDS) gene was co-expressed. GDS catalyzes the condensation of DMAPP and IPP to GPP, which is the substrate for monoterpene synthases [25]. Co-expression of GDS with SfCinS1 in strain AM63-02 (Table 1) led to almost doubling of cineole production compared to the AM63-01 strain. This was further improved when AM63-02 cells were supplemented with exogenous GPP added in the buffer medium (Figure 3C). These results indicated additional potential for higher production levels in the AM63 strain.

### Upregulation of *ERG20 *and haploinsufficiency for *ERG9*

**Figure 4:**
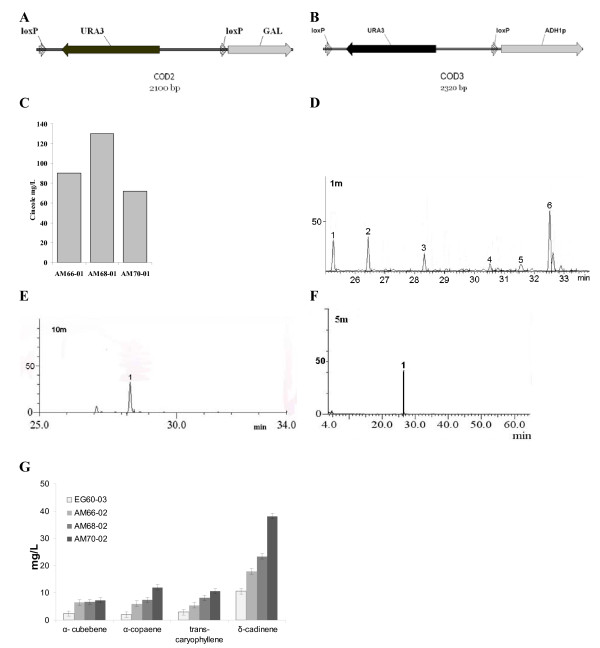
**Improved yeast strains generated by strong promoter integration and single allele deletion for ERG9**. (A) Map of the COD2 cassette, designed for integration of the galactose inducible promoter into promoter regions of endogenous genes. Subsequent to integration, the loxP-URA3-loxP part of the cassette is removed by excision from the enzyme Cre recombinase; (B) Map of the COD3 cassette, designed for the integration of the constitutive ADH1 promoter; C) Cineole production increase in AM68-01 cells, which overexpress ERG20, under the galactose promoter. AM70-01 cells, which are additionally haploinsufficient for ERG9, present substantially reduced levels of cineole production; (D) Expression of a sesquiterpene synthase P330 from *S. pomifera *in AM68-02 cells; peak 1, α-cubebene; peak 2, α-copaene; peak 3, trans-β-caryophyllene; peak 4, δ-cadinene; (E) Trans-β-caryophyllene standard used for quantification (peak 1);. (F) Copaene standard used for quantification (peak 1); (G) Sesquiterpene yields in the newly developed yeast strains. AM70-02 cells exhibit a substantial yield increase compared to wild type EG60-03 cells;

To further modify the biosynthetic pathway, the *ERG20 *gene was chosen as target for upregulation. *ERG20 *encodes for a dual specificity enzyme which is primarily responsible for FPP biosynthesis, but also produces GPP (Figure 2) [26]. Since *ERG20 *is an essential gene in yeast, the diploid strain AM66 was chosen for modification of one of the two alleles. To achieve this, we developed a set recombination cassettes which enable the integration of a strong inducible or constitutive promoter, and the subsequent excision of the selection marker. This strategy enables the consecutive integration of promoter elements at any desired locus. The new cassettes were named COD2 and COD3 (Figure 4A, B). The COD2 cassette was used in a PCR reaction with primers containing *ERG20 *promoter flanking sequence The galactose promoter was then integrated in the promoter region of one of the ERG20 alleles in AM66 cells generating the AM67 strain (Table 1). Verification of the integration site was performed by PCR using primers from the galactose promoter and *ERG20*. Colonies that contained the promoter integration were transformed with the pB227/Gal-cre plasmid expressing the enzyme Cre recombinase under the control of the galactose promoter (Strain AM67-01; Table 1) [27]. Induction of Cre expression led to the excision of the *URA3 *cassette from the genome, leaving behind only the galactose promoter. The final strain AM68-01 (Table 1) was found to have an additional 30% increase in cineole produced (Figure 4C).

An additional means to enhance the availability of isoprene units as substrates for the exogenous plant terpene synthases, is to avoid draining towards sterol synthesis. To this end, *ERG9 *which encodes squalene synthase was targeted. *ERG9 *is the enzyme that acts downstream of ERG20, receiving FPP for its immediate conversion into squalene (Figure 1). Its deletion in haploid cells leads to viability loss [28], but deletion of one allele in diploid cells, has no effect. The haploinsufficient strain for *ERG9 *is expected to express reduced mRNA levels for the gene. To generate such strain a loxP-his5-loxP cassette was employed to delete one of the two alleles in the AM68 strain and generate the strain AM70 (Table 1) [27]. When AM70-01 (Table 1) cells were tested for cineole production, a drastic decrease in cineole production was observed compared to its parental AM68-01 strain (Figure 4B).

Since *ERG20 *produces FPP which is the substrate of sesquiterpene synthases we tested the strains by overexpressing a recently isolated by our group sesquiterpene synthase homologue (P330) from *Salvia pomifera*, into the EG60, AM66, and AM68 and AM70 cells generating EG60-03, AM66-02, AM68-02 and AM70-02 respectively (Table 1). Induction of enzyme expression and analysis of the volatiles identified 4 sesquiterpene peaks (Figure 4E). The highest peak identified, corresponded to *δ*-cadinene, and three almost equivalent peaks corresponded to *trans*-*β*-caryophyllene, *α*-copaene and *α*-cubebene (identified by GC-MS analysis). Quantitation of sesquiterpenes was carried out on the registered peak areas of the detected *trans*-*β*-caryophyllene, as compared to a standard *trans*-*β*-caryophyllene sample (Figure 4F). Sesquiterpene production was increased in all modified yeast strains. Expression of P330 sesquiterpene synthase in AM70 cells, unlike the case for monoterpenes, resulted in a significant increase of sesquiterpenes produced, yielding almost 4-fold increased levels for *δ*-cadinene and *trans*-*β*-caryophyllene (Figure 4D).

### Integration of a promoter-IDI1-ts cassette

**Figure 5:**
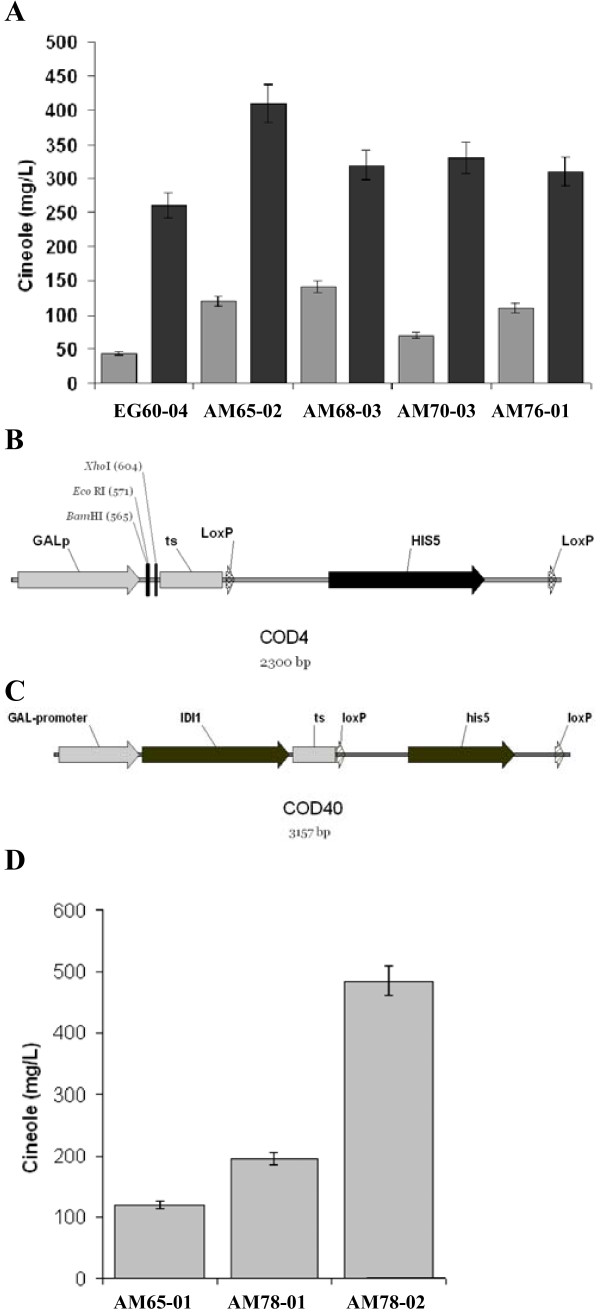
**High levels of IDI1 lead to high monoterpene production**. (A) Co-expression from a plasmid of the yeast *IDI1 *gene in EG60-04, AM65-02, AM68-03, AM70-03, AM76-01 cells (dark bars) leads to substantial increase in cineole yields in all strains tested vs. their corresponding non expressing strains (light grey bars); (B) Map of COD4 cassette which can accept cDNAs under the control of the galactose promoter; (C) Map of COD40 cassette which integrates an extra copy of the *IDI1 *gene into the chromosome, under the control of the galactose promoter. Subsequent to integration, the loxP-his5-loxP part of the cassette is removed by excision from the Cre recombinase; (D) Cineole production in AM78-01 and AM78-02 cells, which have a copy of the P_Gal1_-*IDI1*-ts in the *leu2 *locus.

Τhe gene *IDI1 *(Figure 1) encodes for an isomerase that catalyzes an essential step in the sterol pathway, the isomerization of IPP to DMAPP. The produced DMAPP is then taken up by *ERG20 *to generate GPP. Upon tilting the balance in favor of DMAPP, an increase in GPP and monoterpene production is anticipated. To test this, the open reading frame of *IDI1 *was cloned into the high copy number yeast expression plasmid pYES2-myc under the control of the galactose promoter. *IDI1 *was co-expressed together with SfCinS1 in EG60, AM65, AM68, AM70 and AM76 cells generating the strains EG60-04, AM65-02, AM68-03, AM70-03 and AM76-01 respectively (Table 1). A dramatic increase in cineole production caused by *IDI1 *overexpression, was observed in all strains (Figure 5A). The highest relative increase occurred in the unmodified EG60 cells which reached 5-fold higher levels. The highest absolute levels were produced in the haploid AM65 strain reaching 400 mg/L. Cineole levels at AM68, AM70, and AM76 cells were equivalently high (300 mg/L). To improve the endogenous capacity of our yeast strain to produce GPP, the approach taken was to introduce an extra copy of the *IDI1 *gene into the yeast genome. A new cassette named COD4 (Figure 5B) was developed which included the P_gal1_-MCS-ts- *loxP-his5-loxP*. This cassette can be used to integrate any exogenous gene of interest under the control of the galactose promoter at any desirable locus, and subsequently excise the selection marker and reuse the integration strategy to incorporate the P_Gal1_-GeneX-ts at other loci in the yeast genome [27]. The *IDI1 *orf was cloned into the cassette generating the COD40 construct (Figure 5C).

The 3.1 kb cassette was PCR-amplified using primers that would add 50 bp flanking sequences from the *LEU2 *gene, which is mutated in the parental AM65 strain. Cells which had integrated the COD40 cassette were able to grow in media lacking histidine. The yeast strain was named AM77 (Table 1). The loxP-his5-loxP part of the cassette was subsequently excised using Cre recombinase leaving in the genome only the P_Gal1_-*IDI1*-ts sequence (AM78; Table1). The generated strains were tested for cineole production by expressing SfCinS1 from the pJG4-4 plasmid (Figure 5C). The *IDI1 *integration in strain AM78-01 (Table1) led to a significant increase in cineole yield with production reaching 200 mg/L (AM78-01). These levels are 24-fold higher than the standard reference strain BY4741-02 (Figure 1), although not surprisingly less than the levels attained when *IDI1 *was expressed from a high copy plasmid in the parental strain AM65-02 which attained 400 mg/L (Figure 5A, Table 1). When the plasmid expressing *IDI1 *was introduced to AM78 cells which carried the extra *IDI1 *copy in the genome, the cineole yield reached 485 mg/L surpassing AM65 cells expressing IDI1 (Figure 5A and 5D). These results indicate that by integrating additional copies or resorting to a stronger promoter we can further improve yields at this step. In addition, we tested sesquiterpene production in AM68 and AM70 cells by co-expressing *IDI1 *with P330. Again, unlike monoterpenes, no increase was seen in sesquiterpene production upon *IDI1 *co-expression (data not shown.)

### Robustness of improved yeast strains

**Figure 6:**
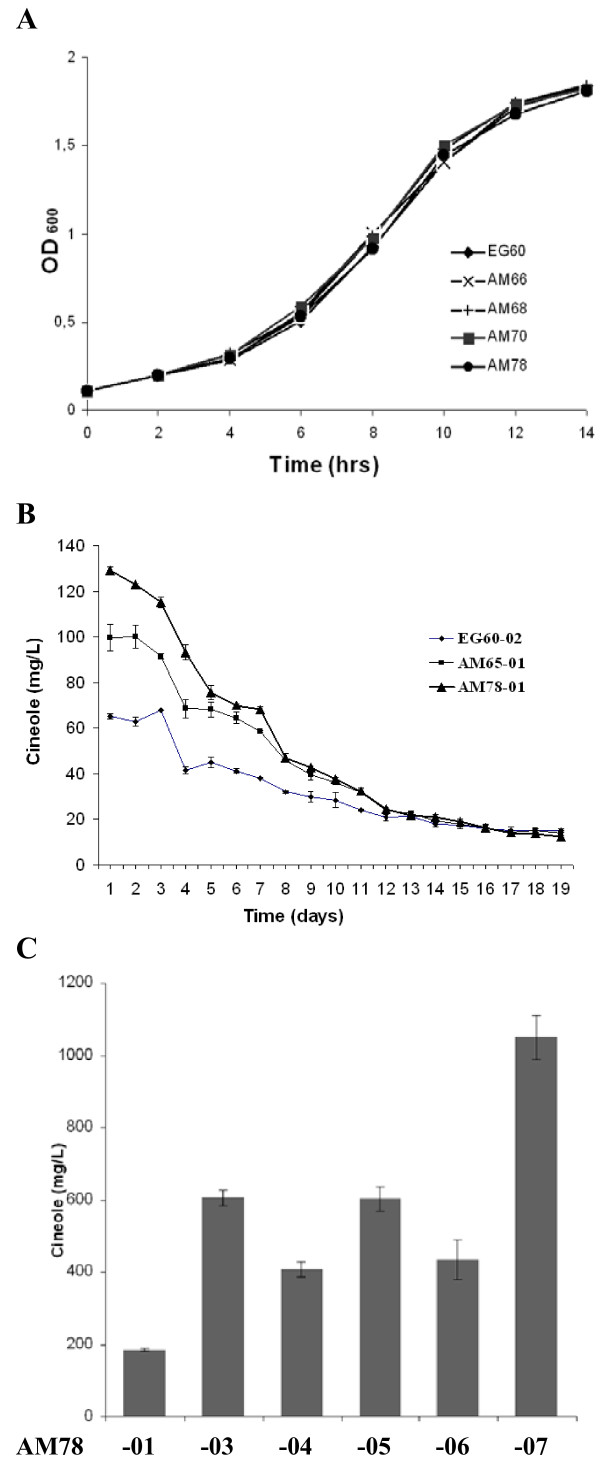
**The new strains are robust and allow further improvements through plasmid-driven gene expression**. (A) The robustness of the newly developed AM66, AM68, AM70, AM78 strains were tested in a growth assay in galactose based media. No difference with wild type EG60 cells is observed; (B) AM78-01 cells (triangle), AM65-01 cells (square), and parental EG60-02 (rhomboid) cells induced for protein expression were sampled by SPME daily over a 19 day period and monitored for cineole production; (C) Co-expression in AM78-05 cells of *IDI1 *from two high copy number plasmids did not cause higher cineole production, whereas the co-expression of *IDI1 *with *HMG2 *(K6R) in AM78-07 doubled the yield.

To assess whether the introduced changes caused growth impediments in the generated strains, fresh overnight cultures of EG60, AM66, AM68, AM70 and AM78 cells were resuspended at low density (OD_600_) in YPD or galactose-raffinose based medium. The cultures were allowed to grow shaking at 30°C and the OD_600 _was measured at regular intervals. No inhibitory effect was observed in any of the tested strains in both media. No changes in cell viability were observed 5 days after induction. (Figure 6A). The results suggest that additional improvements can be undertaken on these strains to maximize yields further.

Additionally, the release of cineole, over an extended time period, was examined. The parental EG60-02 cells together with AM65-01 and AM78-01, harboring the SfCinS1 construct, were grown at mid-logarithmic phase and then switched to galactose-raffinose media for 12 hours to induce expression of the genes. Subsequent to induction the cells were washed and resuspended in buffer solution. The ambient environment of the flasks was sampled daily by SPME. As shown in figure 6B, AM78-01 (-▲-) and AM65-01 (-■-) cells compared to wild type EG60-02 release significantly higher levels over an extended period reaching 12 days post-induction. While the total levels of cineole were lower than in galactose- raffinose media, the release of cineole for all strains was maintained over a longer period.

### Maximizing production in the improved strains by plasmid-driven expression

To further explore the potential of the system, the improved AM78-01 strain expressing SfCinS1, was transformed with combinations of plasmids harboring additional copies of *IDI1 *and *HMG2 *(K6R) or control vectors. This led to the development of the: AM78-03 strain carrying P_Gal1_-*IDI1 *in pYESmyc and the control P_Gal1 _pYIC1 vector; AM78-04 strain carrying the pYESmyc empty plasmid and the P_Gal1_-*IDI1 *in pYIC1; AM78-05 carrying P_Gal1_-*IDI1 *in pYESmyc and P_Gal1_-*IDI1 *in pYIC1; AM78-06 carrying P_Gal1_-*IDI1 *in pYESmyc and P_Gal1_-*HMG2 *(K6R) in pYIC1 (Table 1, Figure 6C).

Production of cineole in strain AM78-05 appeared to reach a plateau compared to AM78-03 strain pointing to saturation at the current balance of the other biosynthetic components. Co-expression of *IDI1 *with the HMG2 (K6R) variant in AM78-06 cells doubled cineole production (Figure 6C). The combination of the two genes expressed from the chromosomal integrations and the high copy plasmids in AM78-07 led to a 55-fold cineole increase from the wild type EG60-02 parental cells (Figure 2D).

### Liquid-Liquid extractions in the improved strains

**Figure 7:**
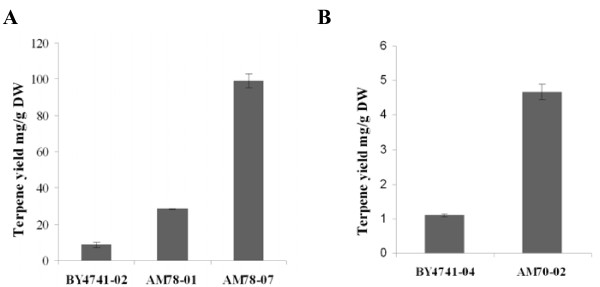
**Terpenoid yields from Hexane/ethyl acetate LL extraction in 3 days old cultures**. (A) A 3.3-fold increase and 11-fold increase of terpenes extracted is observed between AM78-01, AM78-08 respectively compared to control cells. (B) A 2.6-fold increase and 4-fold increase of terpenes extracted is observed between AM68-05, AM78-10 respectively compared to control cells. (C) A 4.2-fold increase in terpenes extracted is observed between AM70-02 cells and BY4741-04 cells expressing the SpP330 sesquiterpene synthase.

The SPME sampling methodology is a powerful technique for the determination of the volatile composition of the head-space above the examined yeast cultures. It allows for the detection of volatiles present in minute amounts, while when combined with signal calibration using standard compounds (see methods) it can provide adequately accurate determination of production yields. However, SPME sampling cannot provide information on the levels of compounds retained in the intracellular space, the cell membrane, or in the medium. To assess these quantities and to provide an SPME-independent evaluation of terpene yields in the improved strains, liquid-liquid extractions were performed. Cultures were induced for 3 days and extracted with an 85:15 mix of hexane/ethyl acetate according to the method described by Takahashi [29]. The extracted material was purified by silica gel column chromatography and the purified fractions were analyzed by GC-MS and quantified. The strain AM78-01 expressing cineole synthase yielded 28.5 mg/g dry weight (DW) of cell mass which was 3.4-fold higher than in control BY4741-02 cells. The strain AM78-07 which additionally expressed *IDI1*and *HMG2 (K6R) *from a plasmid produced 99 mg/g DW, which was 11-fold higher than control cells (Figure 7A). Extraction of sesquiterpenes from SpP330 cells did not exhibit high yields, 4.6 mg/g DW for AM70-02 and 1.1 mg/g DW for BY4741-04. The difference in yield amounts to 4.18-fold (Figure 7B).

The relative proportions of terpenes identified by SPME and by liquid-liquid extraction of cineole producing strains differed. The major compound identified was *β*-pinene reaching 31.59% of the total yield. Limonene and cineole were present in almost equal proportions 13.36% and 12.69% respectively. The SpP330 extraction results gave low yields of only two sesquiterpenes *α*-humulene at 52.06% and *α*-guaine at 35.43%, which were only minor compounds by SPME. These differences likely reflect the differential solubility of the products in an aqueous environment or in the cell membrane. Nevertheless, the yields measured using liquid-liquid extractions confirm the positive effect of the applied genetic modifications in terpene production.

Comparison of the plant essential oil analysis in *S. fruticosa *and *S. pomifera *in respect with the product profiles identified by SPME GC-MS analysis, of SfCinS1 and SpP330 expressing cells, identified a number of common compounds (Table 2) [30-32]. They are present in substantial quantities in the yeast assays. For sesquiterpenes, α-cubebene, caryophyllene, α-humulene and δ-cadinene produced in yeast cells expressing SpP330, are also found in *S. pomifera *plants. The compounds α-copaene, γ-gurjunene and cadina-1,4-diene are encountered in the yeast system but not in plants (Table 2). A number of compounds produced *in planta *though are likely to be further modified by additional enzymes. The improved yeast strains could be helpful in identifying the substrate specificity of enzymes which have been difficult to characterize using in-vitro assays.

**Table 2:** Percentage of mono- and sesquiterpene products produced in plants, in yeast cells, and in enzymatic assays with bacterially expressed protein.

Compounds	*Salvia fruticosa *plants (%)	*Salvia pomifera *plants *(%)*	SfCinS1 SPME (%)	SfCinS1 LLE (%)	SfCinS1 in-vitro (%)	P330 SPME (%)	P330 LLE (%)
**α-Thujene**	0.30 - 1.04	0.24 - 0.84	-	-	-	-	-
**α-Pinene**	2.58 - 6.26	0.42 - 2.40	7.78	16.89	4.6	-	-
**Camphene**	0.17 - 1.05	0.51 - 4.42	-	-	-	-	-
**Sabinene**	0.31 - 0.69	1.00 - 2.65	-	3.14	3.6	-	-
**β-Pinene**	8.92 - 18.77	0.40 - 1.85	8.10	31.59	9.1	-	-
**β-Myrcene**	1.47 - 5.03	0.48 - 1.38	4.9	4.65	2.2	-	-
**α-Phellandrene**	-	-	-	4.16	-	-	-
**Limonene**	0.06 - 1.28	0.27 - 0.59	-	13.36	<1.0	-	12.5
**1,8-Cineole**	22.70 - 49.20	0.09 - 0.20	75.09	12.69	72.4	-	-
**α-Terpinene**	0.38 - 0.80	0.20 - 0.49	3.57	0.33	-	-	-
**γ-Terpinene**				3.33			-
**α-Terpineol**	-	-	0.54	2.22	7.1	-	-
**α-Thujone**	0.30 - 3.53	9.8 - 47.35	-	-	-	-	-
**β-Thujone**	0.22 - 1.72	17.8 - 27.72	-	-	-	-	-
**Camphor**	0.13 - 1.72	0.34 - 1.18	-	-	-	-	-
**Borneol**	0.51 - 1.34	0.14 - 0.93	-	-	-	-	-
**Geraniol**	-	-	-	6.89	-	-	-
**α-Cubebene**	0.26 - 0.19	1.40 - 4.68	-	-	-	10.57	-
**α-Copaene**	-	-	-	-	-	14.45	-
**Caryophyllene**	4.64 - 12.75	7.82 - 22.75	-	-	-	11.25	-
**α-Guaiene**	-	-	-	-	-	1.24	35.43
**α-Humulene**	2.18 - 4.13	0.43 - 1.49	-	-	-	1.86	52.06
**γ-Gurjunene**	-	-	-	-	-	2.40	-
**Germacrene A**	-	-	-	0.41	-	-	-
**Germacrene D**	-	-	-	0.27	-	-	-
**Curcumene**	0.03 - 0.18	0.12 - 5.16	-	-	-	-	-
**Cadinene**	0.03 - 0.70	2.29 - 8.00	-	-	-	50.61	-
**Cadina-1,4-diene**	-	-	-	-	-	4.58	-

### An HSP90 protein isolated from a two-hybrid screen as SfCinS1 interactor enhances cineole production in yeast

**Figure 8:**
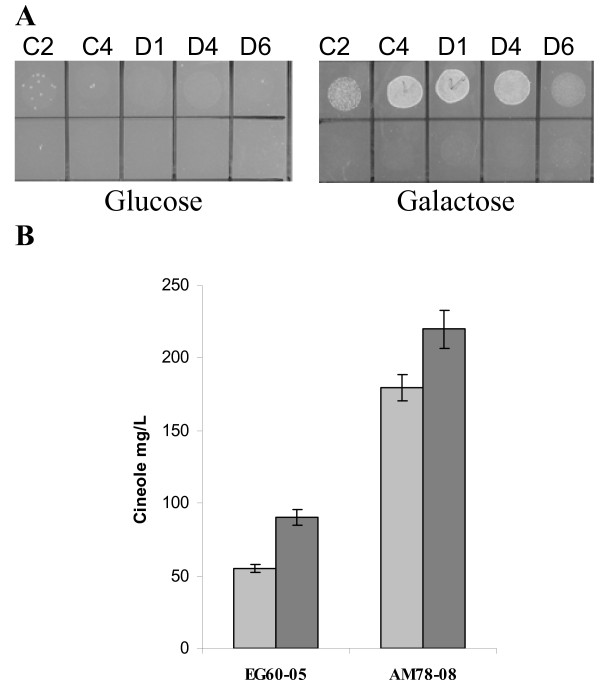
**The putative interacting HSP90 synergizes with SfCinS1 to increase cineole production**. (A) EGY48 yeast reporter cells carrying the SfCinS1-LexA (top row) or the LexA plasmid (bottom row) with the five (C2, C4, D1, D4, D6) library cDNAs were replica plated on glucose/CM-his, trp, leu and galactose-raffinose/CM-his, trp, leu agar media. Growth is observed only for the SfCinS1-LexA fusion in the galactose based media, where proteins are expressed, confirming the specificity of interactions. (B) EG60-05 cells and AM78-08 cells carrying SfCinS1 with empty vector (light grey) and EG60-05 and AM78-09 cells carrying SfCinS1 with HSP90 (dark grey), were tested for cineole production. The presence of HSP90 enhances cineole yield for both strains.

Complementary to the upregulation of the endogenous biosynthetic pathway to increase cineole production, we undertook to examine whether we could enhance terpene production by improving the capacity of cineole synthase. The isolation of terpene synthase interacting proteins, which participate in the folding, stability or localization of the enzyme, could identify additional aspects of the biosynthetic process that could be improved and incorporated into a yeast biotechnological production system approach [33]. To this end, a *S. fruticosa *glandular trichome cDNA library was transferred into a modified pJG4-5 library vector. Additionally a "bait" vector [34] was constructed so as to incorporate the LexA fused to the C-terminus of SfCinS1 to minimize interference of the fused moiety to the enzyme. When tested for cineole production, the SfCinS1-LexA fusion was an active enzyme. The library was introduced into EGY48 yeast reporter cells carrying the SfCinS1-LexA "bait" plasmid. In a pilot screen twenty four colonies were isolated and further processed to extract the library plasmids. Five isolates which were distinctly different in insert cDNA size were tested in fresh cells for the reproducibility of the interactions. The isolated library clones were transformed back into fresh EGY48-01 cells carrying the SfCinS1-LexA fusion and EGY48-02 cells carrying the LexA protein only. Transformed colonies were replica plated in glucose and galactose plates lacking leucine. Growth appeared only in EGY48-01 cells expressing the LexA-SfCinS1 with the putative interactors in galactose plates where proteins are expressed from the P_Gal1 _promoter (Figure 8A).

Three of the isolates C4, D4 and D1 (Figure 8A) passed successfully these tests, as they interacted specifically with the SfCinS1 but not the LexA alone. The cDNAs encode for a luminal binding protein, BiP5 (clone C4), a Heat Shock Protein 90 (clone D4) and a *SEC14 *homologous protein (clone D1). All three cDNAs were subsequently identified as multiple isolates. We focused initially on the HSP90 protein, as chaperones are known to affect the folding of their binding partners. Cineole synthase and the HSP90 protein were co-expressed in EG60-06 parental and AM78-09 improved yeast cells. The presence of HSP90 led to a 60% increase in cineole production in EG60-05 cells, and a 20% improvement in the high cineole producing AM78-08 cells (Figure 8B). The results indicate that proteins potentially interacting with cineole synthase could also augment productivity.

## Discussion

Terpenoids are members of a vast group of secondary metabolites which include sterols, polyprenyl alcohols, carotenoids, ubiquinone (coenzyme Q), and heme A. Commercial interest for them is very high, for their properties as food colorants and antioxidants (carotenoids) [35], aroma and flavor compounds (terpenoids), nutraceuticals (ubiquinone) [36], antiparasitic (artemisinin) [14] and antineoplastic (taxol) agents [3] and biofuels (farnesene, pinene) [5,6]. Although terpenoids are structurally heterogeneous, exhibiting hundreds of different carbon skeletons, they all derive from the universal precursor isopentenyl diphosphate (IPP) and its allylic isomer DMAPP. In yeast these precursors are intermediate products of the mevalonate biosynthetic pathway responsible for sterol biosynthesis. Sterols are important for the physiology of a eukaryotic organism as they form part of the cellular membrane where they modulate their fluidity and function. Ergosterol is the main sterol produced in yeast and is an important constituent of secretory vesicles and for mitochondrial respiration [37,38]. The presence of terpenoid enzymatic precursors, the in-depth understanding of yeast genetics and physiology and the capacity to introduce extensive genetic modifications, attracted early on the focus of research towards biotechnological production of terpenoids in yeast [14,17,39,40]. The approach undertaken presently aimed to develop a system for introducing unlimited successive modifications without the incorporation of undesired bacterial plasmid sequences. The wild type EG60 strain was identified as more capable of producing substantially higher levels of cineole than the standard reference strain BY4741. EG60 cells were previously observed to be very efficient in respiration, to exhibit increased resistance to oxidative stress [41] and produce higher levels of squalene (unpublished data). Extensive variability between various wild type strains was also observed recently by Ohto and co-workers testing ATCC yeast strains for prenyl alcohol production [19].

The enzyme 3-hydroxy-3-methylglutaryl-coenzyme A reductase (HMGR) is of key importance in the mevalonate pathway and is subject to feedback regulation as part of the cellular control of sterol biosynthesis [23]. Yeast has 2 isozymes of HMG-CoA reductase, called hmg1p and hmg2p encoded by *HMG1 *and *HMG2 *genes respectively. Between the two HMG genes in yeast, Hmg1p is quite stable, whereas Hmg2p undergoes sterol pathway degradation [24]. Recently, Garza and colleagues showed that naturally synthesized GGPP controls Hmg2p stability by directly altering the structure of the protein [42]. The choice of the K6R mutation in *HMG2 *aimed to maintain the specific localization of the enzyme, which is mostly perinuclear with intense bead-like foci, while suppressing degradation [43]. Proper localization of the enzyme could be of importance to toxicity, as overexpression of tHmg1 was reported to result in growth properties reduction in yeast strains [44]. Expression of *HMG2 *(K6R) either from a high copy number plasmid or from a genome integrated extra copy did not alter the growth properties of the strains used [44].

The first cassette developed in this work, COD1, incorporated in the HO locus the K6R variant form of *HMG2*, thus making *HMG2 *more stable (Figure 3A). The *URA3 *selection marker was subsequently removed by hisG recombination. This gave rise to strains AM63 and AM65 (haploid) and AM66 (diploid) which produced substantially higher levels of monoterpenes than parental EG60 cells. Since the COD1 cassette, which was based on D. Stillman's vector [20], could only be used for a single integration into the HO locus, we undertook to develop integration vectors that would enable the stable integration of strong promoters or composite inserts (promoter-gene-termination sequence) in any desirable genomic locus. The promoter insertion approach was tested on the *ERG20 *gene which encodes the farnesyl diphosphate synthase, a key enzyme catalyzing the sequential head-to-tail condensations of isopentenyl diphosphate (IPP, C5) with dimethylallyl diphosphate (DMAPP, C5) and GPP to give FPP [26]. Introduction of the strong inducible galactose promoter upstream of one the *ERG20 *allele led to an increase in cineole, as well as sesquiterpene (α-cubebene, α-copaene, trans-caryophyllene and δ-cadinene), production ((Figures 4A and 4G). Further increases of *ERG20 *levels by expressing the gene from a P_Gal1 _plasmid did not lead to any additional monoterpene increases and were mildly toxic (data not shown). Interestingly, decreasing the abundance of *ERG9 *squalene synthase by eliminating one allele caused a decrease in monoterpene production, but a substantial increase in sesquiterpene production. This may be due to FPP accumulation which probably inhibits erg20p through product inhibition, thus lowering the availability of GPP [26,45]. In the AM70-02 cells no such inhibition is observed since the SpP330 enzyme consumes FPP to synthesize sesquiterpenes.

*ERG9 *downregulation has previously been obtained by replacing the endogenous *ERG9 *promoter with a regulatable MET3 promoter, that is repressed in the presence of methionine [15]. Previous observations in allele inactivated diploid strains had shown that in at least 80% of the genes tested this heterozygocity leads to almost 50% reduction in protein levels [46,47]. As anticipated *ERG9*/*erg9 *heterozygocity is shown to cause an increase in sesquiterpene production. This approach may have some merit as it does not require changes in the nutrient medium, which could add an extra level of complexity in a production system. Moreover, it did not cause a growth impediment, and, in principle, additional genes could be targeted for allele inactivation, either in the downstream pathway or in side-pathways draining the substrate supply. In reported strains with downregulated *ERG9 *expressing the sesquiterpene synthase cubebol synthase, farnesol production has been reported to accumulate as resulted of increased FPP availability [44]. No farnesol was detected upon expression of the *S. pomifera *P330 enzyme. On the other hand, the presence of farnesol was manifested when a different sesquiterpene synthase enzyme isolated from *S. fruticosa *was expressed. In the strain with *erg9 *allele inactivation, farnesol comprised almost 50% of the total yield (data not shown).

Shifting the balance towards GPP production was achieved by overexpression of *IDI1*, initially from a plasmid and subsequently by integrating a cassette P_Gal1_-*IDI1*-ts into the mutant *leu2 *locus of the AM65 strain. The strain developed was capable of high monoterpene production while maintaining robustness, thus enabling more changes in the metabolic pathway to further increase production levels. Using the cre-loxP recyclable cassette system no bacterial plasmid sequences are incorporated in the yeast genome, which would not be desirable for certain applications such as production of compounds as food supplements i.e. carotenoids where consumer acceptance is an issue

The central function of a terpenoid synthase is to bind to the flexible isoprenoid substrate and chaperone the orientation and rotational alterations of the substrate and the reaction intermediates. A significant factor contributing to the multitude of terpenoids found in nature is the formation of multiple products. Almost half of all characterized monoterpene and sesquiterpene synthases also form significant amounts of additional products when the expressed protein is assayed in-vitro [48]. This maybe a consequence of high conformational flexibility in the active center, which allows the formation of more reaction intermediates and thus more products. There is no unique feature of the terpene synthases that distinguishes multiple from single product enzymes. Small changes in critical residues can alter dramatically the product profile. Previous work in our lab, showed that the conversion of only five amino acid residues in 1,8-cineole synthase of *Salvia fruticosa*, resulted in switching of the product profile in favor of sabinene as sole product [30]. This is also the case for sesquiterpene synthases. Mutations in four amino acid residues in the active center of *Abies grandis *γ-humulene synthase led to the formation of seven enzymes with different spectra of product specificities [49]. One parameter that should be taken into consideration in the yeast terpene production system is the catalytic capacity of the individual enzyme used. It would be interesting to assess whether mutations could be introduced to the enzymes which would improve their specific activity or increase their product specificity.

We postulated that an additional strategy to increase production of terpenoids in yeast is by co-expressing plant interacting proteins to biosynthetic enzymes. These interactors could facilitate folding of the enzymes, storage and/or secretion of produced compounds. In the pilot screen performed, using a library from glandular trichomes of *Salvia fruticosa*, the three identified proteins are directly relevant to such putative functions. Isolate C4 is a luminal binding protein (BiP), a conserved member of the HSP70 family that is known to bind to incompletely assembled or misfolded proteins in the lumen of the ER [50,51]. Loss of BiP function blocks translocation of secretory proteins in yeast [52]. In plants BiP transcription can be induced by the unfolded protein response. Isolate D1 is homologous to the Arabidopsis SEC14 cytosolic family protein. The SEC14 superfamily consists of greater than 500 members of which the yeast Sec14p is the prototype. Sec14p-like proteins have been adapted during evolution to fulfill a variety of functions that depend on protein-lipid interactions [53]. Isolate D4 which belongs to the family of HSP90-2 proteins was chosen for further characterization. HSP90 proteins are distinct among molecular chaperones in the functionally diverse but numerically restricted substrate proteins which share an inherent conformational instability. Studies have shown the involvement of HSP90 in promoting developmental robustness [54,55]. The HSP90 protein, when overexpressed together with SfCinS1, resulted in a significant production increase confirming the original hypothesis. Co-expression of selected interactors could be an additional strategy in the biotechnological production of terpenoids.

## Conclusion

Yeast strains exhibit high variability in terpenoid production. A suitable wild type strain was selected for improvement. A series of cassettes were developed which allow the integration of any desired genetic element into the yeast genome at suitable loci, avoiding the introduction of unnecessary exogenous DNA sequences. The tools were applied for the generation of new strains with upregulated biosynthesis of terpenoid precursors. The improved yeast strains yielded higher terpenoid levels. Co-expression from high copy number plasmids of *IDI1 *and *HMG2 *(K6R) in the AM78 strain yielded 60% higher monoterpene levels than co-expression in the parental background, indicating that the system is not yet saturated. Additionally, we aimed to stabilize the plant cineole synthase SfCinS1, by co-expressing its interacting partner HSP90, which also led to production increases.

## Methods

### Chemicals and materials

SPME fiber 2 cm-50/30um DVB/Carboxen™/PDMS StableFlex™ Fiber (Supelco);. 1,8 cineole (Aldrich, C8,060-1), γ-terpinene (Aldrich, T2134), α-pinene (Aldrich, P-7408), β-myrcene (M-0382) and (-)-trans-caryophyllene (Sigma, C9653-5), α-copaene (Aldrich, 27814) and a 70% Sabinene solution kindly donated by VIORYL Chemicals, were used as standard compounds. Myc-Tag (9B11) mouse mAb, (#2276, Cell Signaling); HA-Tag (6E2) mouse mAb, (#2367, Cell Signaling) and Anti-Mouse IgG (Fab specific)-Peroxidase antibody (Sigma, A9917) were used for protein detection. Yeast media: D (+)-Glucose monohydrate (16301, Sigma); D-(+) Galactose (G0625, Sigma); Raffinose pentahydrate (R1030, US Biological); Yeast Nitrogen Base w/o AA, carbohydrate & w/AS (Y2025, US Biologicals); Complete Minimal (CM) medium is composed of 0.13% (w/v) dropout powder (all essential amino acids), 0.67% (w/v) yeast nitrogen base w/o AA, 2% glucose. For galactose based medium glucose is substituted with 2% galactose, 1% raffinose; 5'-Fluoroorotic acid monohydrate (F5050, US Biologicals); β-glucuronidase (G7017 Sigma); TOPO TA Cloning Kit Dual Promoter (K4610-20, Invitrogen); SuperSignal West Pico Chemiluminescent Substrate (34077, Thermo Scientific).

### Gene cloning and expression in yeast

The following expression plasmids were used for expression in yeast cells: pYES2 (*URA3*, 2μ, Gal prom.); pYES2-myc (*URA3*, 2μ, P_Gal1_, myc tag); pJG4-4(*TRP1*, 2μ, P_Gal1_); pJG4-6 (*TRP1*, 2μ, P_Gal1_, HA-tag); pJG4-5 (*TRP1*, 2μ, P_Gal1_, B42AD-HA); pYX143 (*LEU2*, ars/cen, P_TPI_); pYX143-HA (*LEU2*, ars/cen, P_TPI_, HA tag); pYES-ADH1 (*URA3*, 2μ, P_ADH1_). The pYIC1 vector was constructed by integration of the *LEU2 *orf, which was PCR amplified using 5'URA-LEU 5'-ACTGCACAGAACAAAAACCTGCAGGAAACGAAGATAAA TCATGTCGCCCCTAAGAAGATCGT-3' and 3'URA-LEU 5'-AGTTTAGTATACAT GCATTTACTTATAATACAGTTTTTTAAGCAAGGATTTTCTTAACTTC -3' primers, into the pYES2 plasmid replacing the *URA3 *marker. The success of recombination was confirmed by PCR and auxotrophic complementation of the new vector. The gene PCR amplifications were performed using Platinum Taq (Invitrogen) or Hotstar Taq (Qiagen) were cloned by TOPO TA cloning into the pCRII vector (Invitrogen). The cloned amplified products were submitted for DNA sequencing prior to additional manipulations. The open reading frame minus the transit peptide (orf) of *S. fruticosa *cineole synthase 1 (SfCinS1, Acc. DQ785793) was PCR amplified from a previously isolated full length cDNA [30] using the primers 5'SfCinS1(+RR) 5'-GAATTCATG CGACGAACTGGAGGCTACCAGC-3' and 3'Sf CinS1(XhoI) 5'-CTCGAGTTAC T CATAGCGGTGGAACAG-3'. The yeast gene isoprenyl diphosphate synthase (YPL117c, Acc. NP_015208) was PCR amplified from yeast genomic DNA using the primers 5'-IDI(EcoRI) 5'-GAATTCATGACTGCCGACAACAATAGTA TGCCCC-3' and 3'-IDI(XhoI)5'-CTCGAGTTATAGCATTCTATGAATTTGCCTGTCAT-3'. The spruce (*Picea abies*) isoprenyl diphosphate synthase PalIDS1 (Acc. GQ369788) was PCR amplified from a full length cDNA, kindly donated by Dr. Gershenzon using the primers 5'GPPS(EcoRI) 5'-GAATTCATGTGCTCAAACACAAATGCCCAG-3' and 3'-GPPS(XhoI) 5'-CTCGAGTCAGTTCTGTCTTTGTGCAATGTA-3'. The *S. pomifera *putative sesquiterpene synthase P330 was PCR amplified from a previously isolated full length cDNA clone (unpublished data) using the primers 5'P330(BamHI) 5'-TGGATCC GAGCTGAAATATATGCATCGGCT-3' and 3'P330 5'-TGATGGAGTTTGATTC TAGACCT-3'. The *Arabidopsis thaliana *HSP90 orf was PCR amplified from the recombinant clone RALF06-10-K09, (RIKEN Arabidopsis) using the following primers: 5'HSP90(*Bam*HI) 5'-GGATCCATGGCGGACGCAGAAACCTT-3' and 3'HSP90(*Xho*I) 5'-CTCGAGGTCAACTTCCTCCATCTTGCT -3'. Expression of the proteins was verified in pYX143-HA, pJG4-6, pYES-myc constructs in western blots using antibodies against the hemaglutinin tag or the myc tag. However, in most cases untagged vector counterparts were used to eliminate the possibility of any interference of the tags.

### Preparation of integration cassettes for yeast transformation

Using genomic DNA from the *S. cerevisiae *strain BY4741 as template, the gene *HMG2 *was PCR amplified with a 5' primer 5'HMG2 (K6R) 5'-ATGTCACTTCCCTTAAGAAC GATAGTACATTTG-3' which introduced a point mutation at K6 converting it to R and a 3' primer 3'HMG2(XhoI) 5'-CTCGAGTTATAATAATGC TGAGGTTTTACAGGGGGG-3'. The PCR product was cloned by TOPO TA cloning (Invitrogen) and sequenced. The galactose promoter (P_gal1_)-multicloning site(mcs)-terminator sequence (ts) was PCR amplified from a yeast expression vector using primers 5'GAL(MfeI) 5'-CAATTGTTTAAACGGATTAGAAGCCGCCGAGCG-3' and 3'CYC1(BglII) 5'-AGATCTGGCCGCAAATTAAAGCCTTCG-3'. The fragment was cloned by TOPO TA cloning and was subsequently transferred into the M4366 vector (Acc. AF324729) generating the construct M4366-Gal1p (HOR-P_gal1_-mcs-ts-hisG-*URA3*-hisG-HOL). The *HMG2 *(K6R) cDNA was excised with EcoRI and the insert was ligated into the M4366-Gal1p vector at the EcoRI site. The orientation of the cDNA relative to the promoter was first assayed using primers from the P_Gal1 _and the 3' of the gene (Figure 3A). The positive construct was additionally validated by DNA sequencing using a primer from the Galp. The full cassette named COD1 (HOR-P_Gal1_-*HMG2 *(K6R)-ts-hisG-*URA3*-hisG-HOL) was linearized by digestion with NotI and was transformed into the EG60 strain. Colonies growing in Glucose/CM-ura were selected and tested for integration using genomic DNA as template and primers from the P_Gal1 _and the *HMG2 *(K6R) cDNA. The strain was named AM63. The *URA3 *selection marker was excised by plating cells on 5-FOA plates which counter-select for the presence of *URA3*. The hisG sequences flanking *URA3 *recombine causing the elimination of the gene. Growing colonies were tested by replica plating on Glucose/CM-ura and were found to be *ura *deficient. A strain of the opposite mating type containing the same COD1 cassette was crossed to AM63 to create a diploid strain homozygous for COD1. The strain was named AM66.

#### Development of COD2 cassette

The galactose promoter was PCR amplified using the primers 5'-GAL(SfiI) 5'-AGTGGCCTATGCGGCCACGGATTAGAAGCCGCCGA-3' and 3'-GAL 5'-AATTCGGATCCAGAGGCATAATCTGGCACATCATACATG-3' which introduce an *Sfi*I site at 5' of the P_Gal1 _promoter. The amplified product was cloned into the pCRII TOPO vector. The construct which contained the SfiI site of the P_Gal1 _proximal to the HindIII site of the vector was used for additional manipulation. The loxP-URA3-loxP fragment from the pUG72 vector was excised by digestion with HindIII and SfiI and inserted into the pCRII- P_Gal1 _plasmid, giving rise to COD2 cassette (loxP-*URA3*-loxP-P_Gal1_). The ADH1 promoter was PCR amplified using the primers SpeI-SfiI 5'ADH 5'-ACTAGTGGCCTATGCGGCCTATTTCGGATATCCTTTTGTTG-3' and 3'- ADH (HindIII) 5'-AAGCTTGGAGTTGATTGTATGCTTG-3' which introduce and SfiI and a HindIII site at the 5' and 3' of P_Adh1 _respectively. The PCR product was cloned and inserted into the pUG72 vector as above, giving rise to the COD3 cassette (loxP-*URA3*-loxP-P_Adh1_.)

To integrate P_Gal1 _upstream of the *ERG20*, using as a template the COD2 cassette the loxP-*URA3*-loxP- *P*_*Gal1*_-HA sequence was PCR amplified by using the following primers: ERG20-COD2-for 5'-CTCAACCAACAGGTATTGGACTGACATAGGCACAATAAACTCAAA AATAAAGCTTCGTACGCTGCAGG-3' and ERG20-COD2-rev 5'-GGGAAAACGTTC AAGAATCTCTCTCTCCTAATTTCTTTTTCTGAAGCCATGGATCCAGAGGCATAAT CT-3'. The products of six PCR amplifications were pooled and purified by phenol/chloroform extraction and ethanol precipitation. The DNA was used in a lithium acetate transformation to transform AM66 cells. Colonies growing in glucose/CM-ura plates were selected and tested for proper integration of the promoter using genomic DNA from the URA+ colonies and primers 5'GALprom and 3' ERG20 confREV 5'-TTGGAAGGTGACCTCATG GAACAATTCG-3'. The PCR products were resolved by agarose gel electrophoresis. Six out of eight tested colonies carried the specific integration. The strain was named AM67. Excision of the URA cassette was achieved by transformation with the plasmid pB227Galp-Cre which expresses the Cre recombinase under the control of the galactose promoter. Cells harbouring the plasmid were plated in Galactose-raffinose/CM-leu media and grown colonies were tested for uracil auxotrophy. The selected *ura *cells were cured of the plasmid and were named AM68.

#### Development of COD4 and COD40 cassettes

The P_Gal1_-mcs-ts was PCR amplified from an expression vector using the primers 5'-GAL (HindIII) 5'-AAGCTTACGGATT AGAAGCCGCCGAG-3' and 3'-ts(SalI) 5'-GTCGACGGCCGCAAATTAAAG CCTTCG-3'. The amplified PCR product was purified by gel extraction and cloned into the pCRII vector in a TOPO TA reaction. The pUG27 vector [27] was modified to destroy the XhoI restriction site. The full cassette was excised using HindIII and SalI and subcloned into the pUG27 modified vector digested with the above enzymes, giving rise to the COD4 cassette. The *IDI1 *orf was inserted into the COD4 plasmid using the EcoRI and XhoI restriction sites found at the mcs generating the COD40 cassette. The previously mutated *leu2 *gene was chosen as the site of integration of the COD4 cassette. The cassette was amplified in PCR reactions using the primers LEU2-pUGF 5'-ATGTCTGCCCCTAAGAAGATCGTCGTTTTGCCAGGT GACCACGTTGGTCAGCTTACGGATTAGAAGCCGC-3' and LEU2-pUG Rev 5'-AG CAAGGATTTTCTTAACTTCTTCGGCGACAGCATCACCGACTTCGGTGGATAGGC CACTAGTGGATCTG-3' and purified as above. The DNA was used to transform AM65 cells. Transformed cells were selected for growth in glucose/CM- his plates. The cells were named AM77.

#### Generation of erg9 haploinsufficient yeast cells

To generate a deletion in one of the two alleles of the diploid AM68 cells, the pUG27 cassette containing the *his5+ *from *S. pombe *flanked by loxP sites was PCR amplified using primers EGR9-pUG F 5'-AGAGAAAAGACGAAGAGCAGAAGCGGAAAACGTATACACGTCACATA TCACAGCTGACTTCGTACGC-3' and ERG9-pUG R 5'-GTACTTAGTTATTGT TCGGAGTTGTTTGTTTATGTTATTTGGCGCAGACTGCATAGGCCACTAGTGGATCTG-3'. AM68 cells were transformed as above and colonies growing in glucose/CM-his. The stable integration of disruption cassette was confirmed by PCR amplification of a 500 bp fragment using the primers ERG9prom 5'-CTAAA CGAGCAGCGAGAACACGACCAC-3' and pUG27confR 5'-GGATGTGATGT GAGAACTGTATCC-3'. The PCR product was resolved by electrophoresis on 1% agarose gel.

### Terpene production in yeast

The developed *S. cerevisiae *strains carrying the plasmids for terpene expression were initially grown in 5 ml overnight cultures in glucose based selective media at 30°C. In the following day 50 ml fresh glucose media was inoculated from the grown cells to an OD_600 _= 0.1 and incubated shaking at 30°C until the OD_600 _reached 0.5-0.7. The cultures were pelleted and washed twice with sterile ddH_2_O. The washed pellets were resuspended in 50 ml of Galactose-Raffinose based selective growth medium and incubated at 30°C ~12 hours. The induced cultures were either kept in the medium and assayed in regular intervals, or washed twice with sterile ddH_2_O and resuspended in 20 ml of buffer (10 mM MOPS, 2 mM MgCl_2_, 0.2 mM MnCl_2_, 2% Glycerol, 1 mM DTT) and incubated at 30°C from 12 hours to several days. When the incubation on buffer was prolonged more than 12 hours the accumulated volatiles were released every 24 hours. Measurements to assess levels of production were performed on cells kept in galactose-raffinose media, while product identification assays and assays for extended periods were performed on cells kept in buffer. Volatiles sampling was performed using a Solid Phase Microextraction (SPME) assembly. The SPME method was performed by using 2 cm-50/30um DVB/Carboxen™/PDMS StableFlex™ Fiber for Manual Sampling. Yeast cells, resuspended in 20 ml of buffer (10 mM MOPS, 2 mM MgCl_2_, 0.2 mM MnCl_2_, 2% Glycerol, 1 mM DTT) and incubated at 30°C, were sampled by exposing the SPME fiber for 30 min into the headspace (using a 250 ml Erlenmeyer flask). 1,8 cineole, γ-terpinene, α-pinene, β-myrcene and (-)-trans-caryophyllene were used as standard compounds. They were dissolved in pentane and ten-fold serial dilutions were prepared. 10 μl from each dilution was added to 20 ml buffer solution and exposed to SPME sampling in identical conditions to the cultures tested, and subsequently measured by GC. The GC cineole peak area values were plotted against the concentration of the compound.

The exposed SPME fiber was withdrawn into the outer septum-piercing needle, removed from the flask, and inserted in the heated injection port of the gas chromatograph. The products were identified by comparing retention times and mass spectra with authentic reference compounds. In-vitro enzyme assays were performed in yeast cell extracts broken by sonication according to the method described by Kampranis et al [30]. The GC apparatus used is a Hewlett Packard 5890 II gas chromatograph equipped with flame ionization detector capillary column HB5 30 m long, with 0.25 mm in diameter and 0.25 μm film thickness. Temperature of injector and detector were 230°C and 270°C, respectively; oven temperature was programmed initially at 60°C for 3 min and then increased with a rate of 3°C/min with a final isotherm at 230°C for 20 min. The carrier gas used for the analysis was Helium (He) at constant pressure of 127 kPa, at 37.1 cm/s velocity. For terpene verification the analytes were thermally desorbed at 230°C from SPME fiber into the injector of a QP2010 Shimadzu gas chromatograph equipped with QP2010 mass selective detector, and a ZB5 (0,25 mm × 30 m, 0,25 μm film thickness) column, in the splitless mode. A further refinement present in the GC/MS is that the coated capillary column is placed in an oven whose temperature is slowly raised with a rate of 3°C/min from 60°C up to 240°C and maintained at this temperature for 5 min to equilibrate. This slow rise in temperature helps to elute compounds with higher boiling points that may not be in gaseous phase in the beginning of the run. Once out of the column the FID will detect the compound.

Liquid-liquid extraction was performed with selected yeast strains which were evaluated for terpene production using 100 ml cultures incubated at 30°C for 3 days. The cultures were extracted with 15% ethyl acetate, 85% hexane [29]. The cell mass concentration was determined by centrifugation of the cell cultures. The collected cell pellet was extensively dried in the oven and weighted. Preparative column chromatography was performed to purify the extracted product, which was concentrated under N_2 _steam to approximately 10% of the original volume and then applied to a silica gel column (5 × 70 mm). Terpenes were then eluted with 90% pentane, 10% diethyl ether in serial fractions. The presence of the products in the separated fractions was confirmed by thin layer chromatography and the identified fractions carrying the products were further analyzed by GC-MS. The amount of purified product was reported to the dry weight (DW) of the corresponding cell pellet.

### Yeast genetic screen for CinS1 interacting proteins

The SfCinS1 PCR amplified insert cloned into the pCRII TOPO vector was digested with EcoRI and XhoI and subcloned into the pGILDA vector linearized with the above restriction enzymes. The composite construct fuses in frame the LexA moiety of the vector with CinS1. Expression of the LexA-CinS1 was verified using antibodies against the LexA part of the fusion protein. A tomato cDNA library cloned into the pJG4-5 vector was screened as previously described [34,56]. Interacting library clones were examined for specificity and submitted for DNA sequencing.

## Abbreviations

SPME: Solid Phase Micro Extraction; CM medium: Complete Minimal medium; FOA: syn. 5'-FOA, 5'-Fluoroorotic acid; HMGR: syn. HMG-CoA reductase, 3-hydroxy-3-methyl-glutaryl-CoA reductase; MVA: Mevalonate pathway; DMAPP: Dimethylallyl pyrophosphate; syn. Dimethylallyl diphosphate; IPP: (3-isopentenyl pyrophosphate; syn. 3-isopentenyl diphosphate; GPP: geranyl pyrophosphate; syn. GDP: geranyl diphosphate; FPP: farnesyl pyrophosphate; syn. FDP: farnesyl diphosphate; GGPP: Geranyl geranyl pyrophosphate; syn. GGDP: Geranyl geranyl diphosphate; ER: Endoplasmic reticulum.

## Competing interests

The authors declare that they have no competing interests.

## Authors' contributions

CI, IC, SL and PK conducted the experimental work. CBJ and SCK assisted in the data analysis and review of the manuscript. AMM conducted some experiments and is responsible for the design and drafting the paper. All authors have read and approved the manuscript.
